# Characterizing and Authenticating Montilla-Moriles PDO Vinegars Using Near Infrared Reflectance Spectroscopy (NIRS) Technology

**DOI:** 10.3390/s140203528

**Published:** 2014-02-20

**Authors:** María-José De la Haba, Mar Arias, Pilar Ramírez, María-Isabel López, María-Teresa Sánchez

**Affiliations:** 1 Department of Bromatology and Food Technology, University of Cordoba, Campus Rabanales, Cordoba 14071, Spain; E-Mails: bt1hacem@uco.es (M.-J.H.); g62armem@uco.es (M.A.); 2 Agricultural Research and Training Centre “Cabra-Priego”, Instituto de Investigación y Formación Agraria y Pesquera (IFAPA), Consejería de Agricultura, Pesca y Desarrollo Rural, Junta de Andalucía, Cordoba 14071, Spain; E-Mails: mariap.ramirez.perez@juntadeandalucia.es (P.R.); mariai.lopez.infante@juntadeandalucia.es (M.-I.L.)

**Keywords:** NIR spectroscopy, wine vinegar, quality parameters, authentication

## Abstract

This study assessed the potential of near infrared (NIR) spectroscopy as a non-destructive method for characterizing Protected Designation of Origin (PDO) “Vinagres de Montilla-Moriles” wine vinegars and for classifying them as a function of the manufacturing process used. Three spectrophotometers were evaluated for this purpose: two monochromator instruments (Foss NIRSystems 6500 SY-I and Foss NIRSystems 6500 SY-II; spectral range 400–2,500 nm in both cases) and a diode-array instrument (Corona 45 VIS/NIR; spectral range 380–1,700 nm). A total of 70 samples were used to predict major chemical quality parameters (total acidity, fixed acidity, volatile acidity, pH, dry extract, ash, acetoin, methanol, total polyphenols, color (tonality and intensity), and alcohol content), and to construct models for the classification of vinegars as a function of the manufacturing method used. The results obtained indicate that this non-invasive technology can be used successfully by the vinegar industry and by PDO regulators for the routine analysis of vinegars in order to authenticate them and to detect potential fraud. Slightly better results were achieved with the two monochromator instruments. The findings also highlight the potential of these NIR instruments for predicting the manufacturing process used, this being of particular value for the industrial authentication of traditional wine vinegars.

## 1. Introduction

Wine vinegar is produced in most Mediterranean countries, and is widely used as a condiment, acidulant and food preservative. The steadily-growing diversity of commercial wine vinegars, coupled with increasing consumer demand, has prompted a need to characterize their major physico-chemical and sensory properties in order to ensure adequate quality control. Quality is governed by the raw material used, the acetification system and, in some cases, the system and type of wood used in the ageing process. Previous research into the monitoring of wine-vinegar quality has been based on a range of chemical and sensory techniques, including pyrolysis-mass spectrometry [1], gas-chromatography-olfactometry [2], atomic absorption spectrometry [3], electronic noses [1] and potentiometry using ion-selective electrodes [4].

Near-infrared reflectance spectroscopy (NIRS) is currently an ideal alternative to traditional wet chemistry for determining vinegar quality. In addition to being fast and precise, it is both flexible and versatile, applicable to multiple products and attributes. A single spectral analysis can provide information on a wide range of quality parameters. This technology is particularly well-suited to the prediction of complex parameters with a crucial influence on end-product quality, such as the ones found in wine vinegars, where information from across the whole spectrum is indispensable.

Recent major advances in NIR instrumentation include an increased use of diode-array equipment varying—amongst other characteristics—in wavelength resolution, detector type, and electronic stability. This has enabled NIRS to be used, with reliable results, for hitherto limited applications; more particularly, it has raised the possibility of on-line use of NIRS technology, although further research is still required in this field.

There has been little research into the use of this technology in vinegar; published papers to date focus mainly on the measurement of total soluble solid content, acidity and pH [5–9]. None of these papers address the comparison of different instruments, with very different specifications in terms of optical design, potential on-line implementation and cost.

Moreover, no use of NIRS technology on the authentication of wine vinegars as a function of the manufacturing method used has been reported to date. However, this is of enormous commercial interest, given the marked difference in quality between traditionally-made vinegars and those obtained using industrial procedures.

The present study sought to investigate the viability of using NIRS technology to evaluate quality parameters in wine vinegars belonging to the Protected Designation of Origin (PDO) “Vinagres de Montilla-Moriles” and to classify wine vinegars according to the method of manufacturing used. At the same time, the performance of three commercial NIRS instruments was compared: two high-end monochromators suitable for laboratory measurements, and a diode-array spectrophotometer suitable for on-line measurements.

## 2. Material and Methods

### 2.1. Sampling

A total of 70 samples of different dry wine vinegars (24 obtained by the traditional “Orleans” acetification procedure and 46 by the submerged culture system), from 12 wineries belonging to the PDO “Vinagres de Montilla-Moriles” were used for this study. Collected samples were transferred to the laboratory at the Bromatology and Food Technology Department, University of Cordoba (Cordoba, Spain), where they were kept refrigerated (2–4 °C) until NIRS spectra were captured the following day. Following spectrum capture, samples were sent to the Agricultural Research and Training Centre in Cabra (Spain) for measurement of eight physico-chemical properties (total acidity, volatile acidity, fixed acidity, pH, ash, dry extract, total polyphenols and color) and to the Agrofood Laboratory and Wine Centre at Jerez (Spain) for measurement of a further three quality parameters (alcohol, methanol and acetoin content).

### 2.2. Analytical Measurements

Total and fixed acidity (g acetic acid/100 mL vinegar) were measured using an automatic titrator (Crison Micro TT 2050, Crison, Alella, Barcelona, Spain) following the official method for Spain [10]. Volatile acidity (g acetic acid/100 mL vinegar) was calculated as total acidity minus fixed acidity. Vinegar pH was measured by potentiometry using the same automatic titrator. Dry extract and ash were measured following the official method for Spain [10], and results were expressed as g/L and percentage point of acetic acid, and g/L, respectively. Acetoin levels (mg/L) were determined by official method for Spain [11], using a gas chromatograph fitted with a flame ionization detector (Thermo-Finnigan Trace GC Ultra, Thermo-Finnigan, Austin, TX, USA). Methanol levels (mg/L) were measured using a Thermo-Finnigan Trace GC gas chromatograph (Thermo-Finnigan) with a capillary column; an internal standard was used for quantitative determination [12]. Total polyphenol content (ppm gallic acid) was measured following [13], using a Perkin Elmer Lambda 25 spectrophotometer (PerkinElmer, Waltham, MA, USA). Color measurements were made using the Sudraud method [14], in which color intensity is taken as the sum of absorbances at 420, 520 and 620 nm and tonality is defined as the ratio of absorbance at 420 nm to the absorbance at 520 nm; the same spectrophotometer was used for this purpose. Alcohol content (% vol.) was measured using an FTIR interferometer (Winescan FT 120, Foss Electric, Hillerød, Denmark) [15]. All analyses were performed in duplicate.

### 2.3. Instruments and Spectrum Collection

For collecting NIR spectra, three NIR-instruments—differing mainly in the wavelength range used and the measuring principle involved—were used. The main features of these instruments are shown in Table 1. Samples were scanned using a folded-transmission gold reflector cup, diameter 3.75 cm, with a pathlength of 0.1 mm, in the transflectance mode, with all three instruments tested.

The FOSS-NIRSystem 6500 SY-I (FNS-I) and the SY-II (FNS-II) monochromators (Silver Spring, MD, USA) provide absorbance readings from 400 to 2,500 nm, in 2 nm steps. The FNS-I instrument is equipped with manual gain control detectors, and a spinning module was used. The later-generation FNS-II is equipped with autogain control, and the transport module was used for spectrum collection. In both cases, two spectra were collected per sample and were averaged for subsequent processing. Spectral data were recorded using the WinISI II software package version 1.50 (Infrasoft International, Port Matilda, PA, USA).

Spectra were also collected on all samples using the Zeiss Corona portable diode-array spectrophotometer (model Corona 45 VIS/NIR, Carl Zeiss Inc., Thornwood, NY, USA) in the spectral range 380–1,700 nm, every 2 nm. The instrument was equipped with the turnstep module (revolving plate) containing the gold reflector cup. Two spectra were captured per sample and the average of the two was used in calculations. For this instrument, the signal was captured using CORA software version 3.2.2 (Carl Zeiss Inc.), and subsequently pretreated using the Unscrambler program version 9.1 (CAMO, ASA, Oslo, Norway).

### 2.4. Calibration Set

Calibration models were constructed using all the samples available (*n* = 70) for all parameters except alcohol content (*n* = 41), since the reference method to measure alcohol content only detects values greater than 0.2% vol. (Table 2).

### 2.5. Chemometric Data Treatment

The WinISI software package version 1.50 (Infrasoft International.) was used for the chemometric treatment of data. Quantitative calibrations were developed for predicting total acidity, fixed acidity, volatile acidity, pH, dry extract, ash, acetoin, methanol, total polyphenols, color, and alcohol content. Prediction equations were obtained using Modified Partial Least Squares (MPLS) as regression method [16] with cross-validation; the calibration set was partitioned into six groups; each group was then validated using a calibration developed on the other samples; finally, validation errors were combined to obtain a standard error of cross-validation (SECV).

For each analytical parameter, different mathematical treatments were evaluated for scatter correction, including the Standard Normal Variate (SNV) and Detrending (DT) methods. Furthermore, four derivate mathematical treatments were tested in the development of NIRS calibrations: 1,5,5,1; 2,5,5,1; 1,10,5,1 and 2,10,5,1, where the first digit is the number of the derivative, the second is the gap over which the derivative is calculated, the third is the number of data points in a running average or smoothing, and the fourth is the second smoothing [16].

For calibration purposes, the following spectral regions were tested: (1) VIS+NIR (500–1690 nm Corona 45 VIS/NIR and 400–2500 nm Foss NIRSystems); (2) only NIR (1100–1690 nm Corona 45 VIS/NIR and 1100–2500 nm Foss NIRSystems). To eliminate signal noise in the diode array instrument at the beginning and end of the spectrum, the wavelength ranges between 380–500 nm and 1690–1700 nm were discarded.

The statistics used to select the best equations were: standard error of calibration (SEC), coefficient of determination of calibration (*R*^2^), standard error of cross-validation (SECV), coefficient of determination for cross-validation (*r*^2^), RPD or ratio of the standard deviation of the original data (SD) to SECV, and the coefficient of variation (CV) or ratio of the SECV to the mean value of the reference data for the calibration set. These latter two statistics enable SECV to be standardized, facilitating the comparison of the results obtained with sets of different means [17].

### 2.6. NIRS Classification Models

The design of models to classify wine vinegar by manufacturing method, in order to evaluate the viability of using NIRS technology for authenticating wine vinegars, comprised two classification groups: traditionally-produced vinegars in which acetic fermentation takes place slowly on the surface of wood barrels, i.e., the traditional “Orleans” acetification process; and industrial vinegars made using the submerged culture process.

The influence of potential imbalance on the development of discriminant models was also investigated using two different models for each of the three instruments tested: a class-balanced model containing 24 samples each of traditional and submerged-culture vinegars; and a class-unbalanced model containing 46 submerged-culture samples and 24 traditional vinegar samples.

Samples for balanced training sets were selected using the SELECT algorithm included in the WinISI II version 1.50 software package, which detects samples whose spectrum is similar to that of others in the population [16].

Discriminant models were constructed to classify wine vinegar by manufacturing method, using PLS discriminant analysis (PLS-DA) for supervised classification. Specifically, the PLS2 algorithm was applied, using the “Discriminant Equations” option in the WINISI version 1.50 software package.

All models were constructed using six cross-validation groups (i.e., the calibration set is partitioned into six groups; each group is then predicted using a calibration developed on the other samples), in the wavelength ranges: (1) 400–2,500 nm, for the FNS-6500 instruments; and (2) 500–1,690 nm for the Corona 45 VIS/NIR. To eliminate signal noise in the diode array instrument at the beginning and end of the spectrum, the wavelength ranges between 380–500 nm and 1,690–1,700 nm were discarded. A combined Standard Normal Variate (SNV) and Detrending (DT) method was used for scatter correction. First and second-derivative treatments were tested: 1,5,5,1; 1,10,5,1; 2,5,5,1 and 2,10,5,1 [16]. The precision of the models obtained was evaluated using the percentage of correctly-classified samples, both globally and partially or by classes.

## 3. Results and Discussion

### 3.1. Overview of Wine Vinegar Spectra

**Figure 1.**
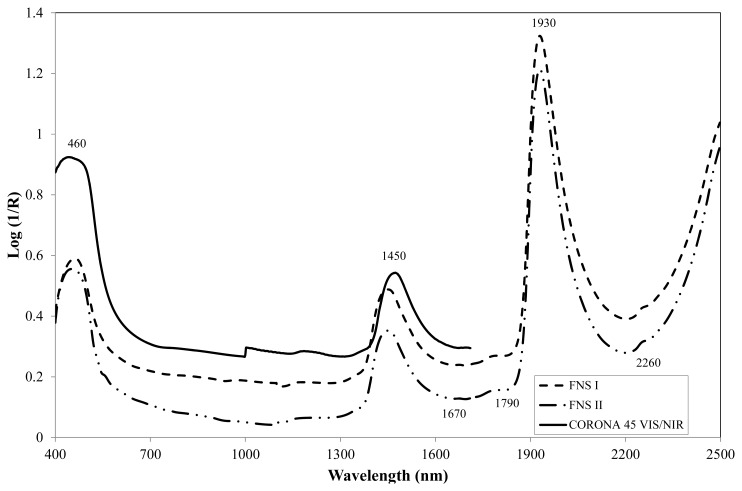
Typical log (1/*R*) spectra for Montilla-Moriles PDO vinegars.

Typical log (1/*R*) spectra for wine vinegars collected using the three instruments tested, together with the most relevant absorption bands, are shown in Figure 1. All spectra displayed fairly similar trends.

One peak was identified in the visible region of the spectrum, at 460 nm, which is indicative of the presence of red pigments (anthocyanins) [18].

In the near-infrared region, all spectra displayed intense bands at 1,450 nm, related to the first O-H overtone, and at 1,930 nm, related to the combination of stretch and deformation of the O-H group in water [19]. The small absorption band at 1,670 nm might be related to the C-H_3_ stretch first overtone or C-H groups in aromatic compounds [20]. The band at 1,790 nm may be related to the C-H stretch first overtone, and that at 2,260 nm probably to C-H combination bands of methanol [21,22].

### 3.2. Calibration Development

The final equations for each parameter studied were selected by statistical criteria, choosing those which displayed the lowest values for SECV and CV, and the highest values for *r*^2^ and RPD. Table 3 shows the cross-validation statistics for the best equations obtained for the three instruments tested.

#### 3.2.1. Acidity-Related Parameters

The best calibration models obtained using the global set (*n* = 70) for the prediction of total acidity, fixed acidity, volatile acidity and pH for the three instruments tested are shown in Table 3.

The equation displaying the greatest predictive capacity for total acidity was that obtained using the FNS-I instrument over the broadest spectral range, i.e., 400–2,500 nm, and with scatter correction. Performance statistics were *r*^2^ = 0.99, SECV = 0.25 and RPD = 8.35. Inferior results were obtained with the FNS II (RPD = 7.95) and with the Corona 45 VIS/NIR (RPD = 7.44). Results for the FNS-I were similar to findings reported by Fan *et al.* [7] and by Chen *et al.* [6], who recorded *r*^2^ values of 0.97–0.99 and SECV values of 0.15–0.25, respectively.

For fixed acidity, the equation displaying the greatest predictive capacity was also obtained with the FNS-I over the range 1100–2,500 mm with scatter correction, yielding statistical values of *r*^2^ = 0.79, SECV = 0.04 and RPD = 2.19; these values were similar to those obtained with the FNS-II (RPD = 2.15) and higher than those yielded by the Corona 45 VIS/NIR (RPD = 1.53). This predictive capacity surpassed that recorded by Sáiz-Abajo *et al.* [5], who reported an RMSEC value of 0.79.

The best equations for volatile acidity were also obtained using the FNS-I, which yielded performance statistics (*r*^2^ = 0.98; SECV = 0.25 and RPD = 7.99) similar to those recorded by González-Sáiz *et al.* [23] in onion vinegar and by Wang *et al.* [24] in plum vinegar, while statistics for pH prediction (r^2^ = 0.85; SECV = 0.05 and RPD = 2.60) were lower than those reported by Bao *et al.* [8]. The predictive capacity of the models constructed using the FNS-II was similar (RPD = 7.16 and 2.25 for the two parameters tested), whilst the performance statistics for the Corona 45 VIS/NIRS were lower for volatile acidity (RPD = 6.92) and for pH (RPD = 1.89).

The overall equations developed with the FNS-I for total and volatile acidity yielded a coefficient of determination (0.99–0.98) that enabled samples to be classed with total accuracy due to the excellent predictive capacity of the model. For fixed acidity and pH, the predictive capacity of the models developed may be considered good according to Williams [17].

Although the best results in terms of predictive capacity were obtained using the FNS-I instrument, the precision and accuracy of the models developed using the Corona 45 VIS/NIR diode-array instrument would amply justify their on-line use for monitoring vinegar fermentation and controlling volatile acidity during the fermentation process, as well as for authenticating wine vinegars, since the measurement of non-volatile acidity attributable to the major fixed acids in wine vinegar (tartaric, malic and succinic) enables them to be distinguished from alcohol vinegars. Similarly, the volatile acidity/dry extract ratio can be used to determine whether the vinegar has been fermented; a low ratio, generally below 8, is recorded for wine vinegars, whereas synthetic, alcohol-based vinegars display a high ratio (10–100).

#### 3.2.2. Parameters Related to Fraud Detection and Vinegar Authentication

The best calibration models obtained for predicting parameters useful for detecting fraud within the wine industry (dry extract, ash, acetoin and methanol content) are shown in Table 3. All three instruments displayed great predictive capacity for dry extract. Results for the FNS-I and FNS-II were very similar (*r*^2^ = 0.99, SECV = 0.14 and 0.12; RPD = 8.16 and 9.41, respectively), scanning over the range 400–2500 nm with the FNS-I and over the 1,100–2,500 nm range with the FNS-II, with scatter correction in both cases; these results were better than those obtained using the Corona 45 VIS/NIR (*r*^2^ = 0.94; SECV = 0.18; RPD = 4.14) over the spectral range 1100–1690 nm without scatter correction. Better results were reported by Sáiz-Abajo *et al.* [5], although they used a larger range for the calibration set (1.30–17.56 *vs.* 0.72–6.71).

Slight differences in accuracy were noted for the ash-prediction models between all three instruments. The best calibration statistics for the FNS-I were *r*^2^ = 0.91, SECV = 0.41, RPD = 3.28; for the FNS II the best values were *r*^2^ = 0.95, SECV = 0.30, RPD = 4.59; and for the Corona 45 VIS/NIR *r*^2^ = 0.83, SECV = 0.55, RPD = 1.62. For the monochromators, the best calibration statistics were recorded using the spectral range 1,100–2,500 nm, whilst the diode-array instrument yielded the best statistics in the range 500–1,690 nm, with scatter correction in all cases.

The predictive capacity of the equations obtained here for ash measurements was lower than the SEC = 0.21 reported by Sáiz-Abajo *et al.* [5].

The predictive capacity of the models constructed to predict acetoin using the FNS-I monochromator (400–2500 nm and scatter correction) (*r*^2^ = 0.71; SECV = 127.33; RPD = 1.86) may be considered good, whilst the models obtained using the FNS-II (400–2,500 nm and scatter correction) and the Corona 45 VIS/NIR (500–1,690 nm and no scatter correction) would enable acetoin values for vinegars to be classified as high, medium, or low (*r*^2^ = 0.56–0.59; SECV = 158.82–155.92; RPD = 1.51–1.56, respectively), following Williams' recommendations [17].

Models constructed for methanol determination using the FNS-II over the whole instrument range, with scatter correction, were more accurate and precise (*r*^2^ = 0.80; SECV = 7.81; RPD = 2.21) than those obtained with the Corona 45 VIS/NIR (RPD = 1.85) and with FNS-I (RPD = 1.73) (Table 3).

No references have been found in the literature to the measurement of acetoin or methanol using NIRS technology.

Calibrations using the two monochromators displayed excellent predictive capacity for dry extract and ashes; predictive capacity was excellent for dry extract and good for ashes using the diode-array instrument; good for acetoin using the FNS-I and fair using the FNS-II and Corona 45 VIS/NIR; and good for methanol using the FNS-II and the Corona 45 VIS/NIR and fair for the FNS-I [17]. This confirms the viability of NIRS technology for detecting vinegar frauds, using this non-destructive technique to increase sampling pressure, and also for on-line monitoring of these parameters; on-line implementation of the diode-array instrument is wholly viable and would provide the required real-time values for monitoring purposes. Dry extract values below the legally-established limits may be an indication that the vinegar has been subjected to unacceptable (or indeed banned) by law treatments, including watering-down and the addition of sugars, alcohol or acetic acid, even though the initial degree of acidity is maintained. By contrast, dry extract values above the regulatory limits may indicate the presence of alcohol fermentation other than the normal acetification process, or excessive crushing or pressing of the grape. The addition of non-volatile substances such as glycerin would also prompt an increase in the figure for dry extract [25]. In the case of ash, heavy dilution of vinegar followed by the addition of mineral acetic acid would be reflected in an abnormally high value for ash content. The non-destructive determination of acetoin or acetylmethylcarbinol—a characteristic compound that accumulates during the acetification process—would greatly help to distinguish genuine fermented vinegars from artificial vinegars, in which this compound is not found [26].

With regard to methanol, since each product has a unique volatile composition, the volatile fraction of different vinegars has been used to distinguish and classify these products, thus helping to combat fraud and ensure the authenticity and quality of wine vinegars [27,28].

#### 3.2.3. Total Polyphenols and Color

The best prediction models for total polyphenols and color intensity and tonality were obtained over the VIS + NIRS wavelength range, using scatter correction (except for total polyphenols and tonality using the FNS-II). As Table 3 shows, the best models for predicting total polyphenol content were obtained with the FNS-I, whose predictive capacity may be regarded as good (*r*^2^ = 0.82; SECV = 56.69; RPD = 2.35) [17]; its performance was similar to that of the FNS-II (RPD = 2.28), and better than that of the Corona 45 VIS/NIR (RPD = 2.14). These results confirm the viability of NIRS technology for predicting a key parameter in the vinegar industry, since phenols are associated with organoleptic properties of color, flavor and astringency in vinegar [29]. Low phenol compound content, taken in conjunction with the results for other quality parameters, may indicate that a vinegar is of inferior quality.

As Table 3 shows, the models best predicting color-related parameters (intensity and tonality) were obtained using the FNS-II instrument, (*r*^2^ = 0.98 and 0.90; SECV = 0.23 and 0.41; RPD = 6.86 and 3.13, respectively) with excellent predictive capacity according to Williams [17]. Poorer results were obtained with the FNS-I (RPD = 4.96 and 2.68) and the Corona 45 VIS/NIR (RPD = 5.88 and 4.19) for intensity and tonality, respectively. The tonality (hue) of a vinegar reflects the ratio of yellow to red coloring. Low values for tonality thus indicated that the vinegar has been made from darker wines. It should also important to consider that contact with wood also prompts a decrease in this value. In the case of intensity, higher intensity values mean that the vinegar has been made from darker wines.

No references have been found in the literature to the prediction of total polyphenol content or color in vinegars using NIRS technology.

#### 3.2.4. Alcohol Content in Relation to the Ageing Process

The best models for alcohol content (*r*^2^ = 0.99; SECV = 0.04; RPD = 22.31) were obtained for the FNS-II in the spectral range 400–2500 nm and without scatter correction (Table 3). The value obtained for *r*^2^ (0.99) would, according to the guidelines indicated by Williams [17], demonstrate the robustness and power of the calibration models. The results obtained with the FNS-I (RPD = 14.42) and Corona 45 VIS/NIR (RPD = 4.98) confirm the accuracy and precision of the models. On-line monitoring of alcohol content would enable the optimum level of residual alcohol at the end of the acetic fermentation process to be clearly established, since exhaustion of alcohol during fermentation is unprofitable, given that in the absence of alcohol substrate acetic bacteria may degrade the acetic acid produced. In traditional manufacturing methods, which generally involve a subsequent period of ageing in wooden casks, complete exhaustion of ethanol is to be avoided, since the aim is to trigger esterification reactions between the alcohol and the acetic acid, which improve the vinegar's bouquet, leading to the formation of esters such as ethyl acetate [25]. Similar results for the prediction of alcohol content have been reported for rice wine by Yano *et al.* [21] and for onion vinegar by González-Sáiz *et al.* [23].

### 3.3. Discriminant Analysis

The results obtained for the best classification models for predicting manufacturing method, using the PLS2-DA algorithm and the three NIRS instruments tested, are shown in Table 4, both for unbalanced and balanced sets. The percentage of correctly-classified samples ranged from 92.8% to 94.3% for unbalanced sets, and from 91.7% to 93.7% for balanced sets.

In unbalanced sets, the most accurate models were obtained using D1 log (1/*R*) for all the instruments tested, whilst for balanced sets the best performance was recorded using D1 log (1/*R*) (FNS-I) and D2 log (1/*R*) (FNS-II and Corona 45 VIS/NIR).

The FNS-I correctly classified 94.3% of vinegars in unbalanced sets and 91.7% in balanced sets, while correct classification rates for the FNS-II were 92.8% and 93.7% and for the Corona 45 VIS/NIR 94.3% and 93.7%, respectively. Similar minimal differences in classification rates regardless of set size have also been reported by Pérez-Marín *et al.* [30], who note that PLS2 is less sensitive to the use of class-unbalanced sets.

Although all models adequately classified wine vinegars belonging to the PDO “Vinagres de Montilla-Moriles” by manufacturing method—indicating that NIR spectra enable discrimination between traditional fermentation, addressed to the production of high-quality vinegars commanding a high price premium in the market, since a very long period of time is required to attain a high acetic degree, and the industrial submerged fermentation method where all the alcohol is turned into acid and a sharper-tasting vinegar is produced [31]—better results were obtained using the Corona 45 VIS/NIR spectrophotometer.

**Figure 2.**
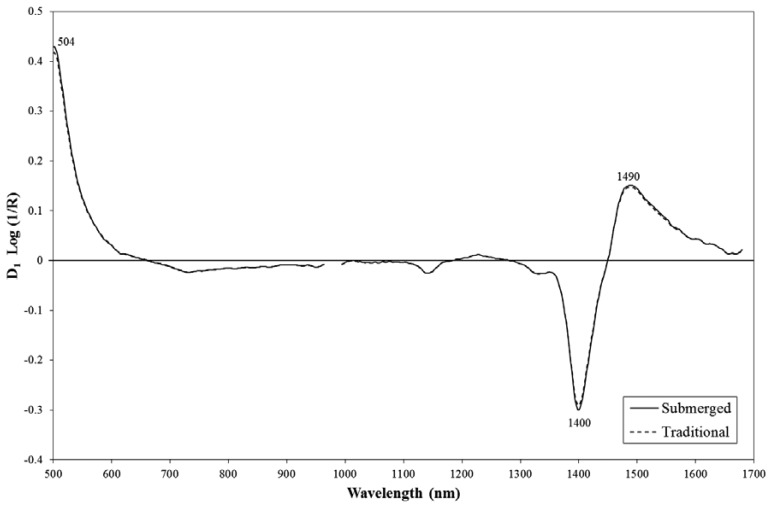
D1 log (1/*R*) spectra for Montilla-Moriles PDO vinegars made by traditional and submerged methods. Corona 45 VIS/NIR spectrophotometer. Spectral range 500–1,690 nm.

D1 log (1/*R*) spectra for wine vinegars manufactured using the traditional and submerged culture systems, obtained by the Corona 45 VIS/NIR instrument, are shown in Figure 2; areas of maximum difference, which are useful for discrimination purposes, are also indicated.

Absorption peaks at 504 nm (mainly linked to anthocyanins), and at 1,400 and 1,490 nm (related to water content and O-H combinations) appear to be especially relevant for the classification of wine vinegar by traditional *vs.* submerged methods. Similar findings were reported by Guerrero *et al.* [32] in a study using traditional analytical techniques to classify vinegars by manufacturing method.

No references have been found in the scientific literature to NIRS-based models for classifying wine vinegars by manufacturing method. However Pizarro *et al.* [33] used a monochromator operating in the spectral region between 1100 and 2500 nm to classify vinegars as a function of both raw material (grape, wine or malt) and ageing period; 100% of samples were correctly classified.

## 4. Conclusions

The models developed here for predicting vinegar quality parameters and authenticating manufacturing methods highlight the potential of NIRS technology as a non-destructive tool for quality control and traceability testing in the vinegar industry. This technology can be used to establish whether a product is genuine, to determine the method by which it was made and to ensure that it meets the legal requirements for PDO “Vinagre de Montilla-Moriles” wine vinegars. Moreover, the results obtained using a latest-generation diode-array instrument were comparable to those obtained with high-end monochromators; the diode-array instrument has the advantage that it can be built into a production line, thus facilitating real-time decision-making throughout the manufacturing process.

## Figures and Tables

**Table 1.** Basic technical characteristics of three spectrophotometers: two monochromators (M) and a diode-array instrument (DA).

**Properties**	**Instruments**	

	**M: FNS 6500 SY-I and FNS 6500 SY-II**	**DA: Corona 45 VIS/NIR**
Detector type	Silicon, 400–1,100 nm. Lead Sulphide, 1,100–2,500 nm	Silicon, 400–950 nm. Indium-Gallium-Arsenide detector, 950–1,700 nm
Wavelength range (nm)	400–2,500	380–1,700
Spectral data rate	1.8 scans/s	20 scans/s
Dispersion	Pre	Post
Light source	Full spectrum	Full spectrum
Analysis mode	Reflectance	Reflectance

**Table 2.** Statistical analysis of calibration sample sets, i.e., data ranges, means and standard deviations (SD) and coefficients of variation (CV).

**Parameter**	**N**	**Range**	**Mean**	**SD**	**CV (%)**
Total acidity (g acetic acid/100 mL vinegar)	70	4.65–15.75	9.44	2.14	22.70
Fixed acidity (g acetic acid/100 mL vinegar)	70	0.01–0.53	0.26	0.09	34.62
Volatile acidity (g acetic acid/100 mL vinegar)	70	4.39–14.81	9.19	2.10	22.87
pH	70	2.49–3.33	2.82	0.15	5.15
Dry extract (g/L and percentage point of acetic acid)	70	0.72–6.71	2.36	1.15	48.81
Ash (g/L)	70	1.40–8.25	3.32	1.42	42.68
Acetoin (mg/L)	70	144.75–2057.17	498.51	336.15	67.43
Methanol (mg/L)	70	12.18–122.74	49.85	21.28	42.69
Total polyphenols (ppm gallic acid)	70	73.39–801.05	344.29	141.08	40.98
Intensity	70	0.03–9.44	1.53	1.89	123.73
Tonality	70	0.10–4.53	2.56	1.30	50.88
Alcohol content (% vol.)	41	0.22–4.70	1.07	0.99	92.04

**Table 3.** Calibration statistics for quality parameters in wine vinegars.

**Parameter**	**Instrument**	**Mathematic treatment**		**Spectral range (nm)**	**Mean ^1^**	**SD ^2^**	**SECV ^3^**	*r* **^2^**,**^4^**	**RPD ^5^**	**CV ^6^**
Total acidity (g acetic acid/100 mL vinegar)	FNS I	2,5,5,1	SNV + DT	400–2500	9.49	2.07	0.25	0.99	8.35	2.62 *
	FNS II	1,5,5,1	None	400–2500	9.50	2.08	0.26	0.98	7.95	2.76
	Corona	1,5,5,1	None	500–1690	9.39	2.17	0.29	0.98	7.44	3.12
Fixed acidity (g acetic acid/100 mL vinegar)	FNS I	1,10,5,1	SNV + DT	1100–2500	0.24	0.08	0.04	0.79	2.19	15.62 *
	FNS II	1,10,5,1	SNV + DT	1100–2500	0.25	0.09	0.04	0.78	2.15	16.70
	Corona	2,5,5,1	SNV + DT	500–1690	0.25	0.08	0.05	0.58	1.53	19.77
Volatile acidity (g acetic acid/100 mL vinegar)	FNS I	2,5,5,1	SNV + DT	400–2500	9.24	2.03	0.25	0.98	7.99	2.76 *
	FNS II	1,5,5,1	None	400–2500	9.11	2.10	0.29	0.98	7.16	3.22
	Corona	1,5,5,1	None	500–1690	9.10	2.13	0.31	0.98	6.92	3.38
pH	FNS I	1,5,5,1	None	1100–2500	2.82	0.14	0.05	0.85	2.60	1.85 *
	FNS II	1,5,5,1	None	400–2500	2.81	0.12	0.05	0.81	2.25	1.95
	Corona	1,5,5,1	None	500–1690	2.80	0.13	0.07	0.72	1.89	2.43
Dry extract (g/L and percentage point of acetic acid)	FNS I	2,10,5,1	SNV + DT	400–2500	2.33	1.13	0.14	0.99	8.16	5.94
	FNS II	1,5,5,1	SNV + DT	1100–2500	2.32	1.12	0.12	0.99	9.41	5.14 *
	Corona	1,5,5,1	None	1100–1690	2.10	0.75	0.18	0.94	4.14	8.65
Ash (g/L)	FNS I	1,5,5,1	SNV + DT	1100–2500	3.26	1.33	0.41	0.91	3.28	12.49
	FNS II	1,10,5,1	SNV + DT	1100–2500	3.28	1.37	0.30	0.95	4.59	9.10 *
	Corona	2,10,5,1	SNV + DT	500–1690	3.29	1.35	0.55	0.83	1.62	16.71
Acetoin (mg/L)	FNS I	2,5,5,1	SNV + DT	400–2500	439.26	237.45	127.33	0.71	1.86	28.99 *
	FNS II	1,10,5,1	SNV + DT	400–2500	436.34	239.58	158.82	0.56	1.51	36.40
	Corona	2,10,5,1	None	500–1690	459.69	244.56	155.92	0.59	1.56	33.92
Methanol (mg/L)	FNS I	2,10,5,1	None	1100–2500	46.94	17.04	9.85	0.67	1.73	20.98
	FNS II	1,5,5,1	SNV + DT	400–2500	46.64	17.25	7.81	0.80	2.21	16.74 *
	Corona	2,5,5,1	None	500–1690	44.35	14.67	7.93	0.71	1.85	17.87
Total polyphenols (ppm gallic acid)	FNS I	1,5,5,1	SNV + DT	400–2500	337.08	133.35	56.69	0.82	2.35	16.82 *
	FNS II	1,10,5,1	None	400–2500	337.08	133.35	58.42	0.81	2.28	17.33
	Corona	2,5,5,1	SNV + DT	500–1690	337.99	136.33	63.58	0.78	2.14	18.81
Intensity	FNS I	1,10,5,1	SNV + DT	400–2500	1.31	1.55	0.31	0.96	4.96	23.93
	FNS II	2,10,5,1	SNV + DT	400–2500	1.48	1.66	0.23	0.98	6.86	16.41 *
	Corona	1,5,5,1	SNV +DT	500–1690	1.31	1.56	0.27	0.97	5.88	20.32
Tonality	FNS I	2,10,5,1	SNV + DT	400–2500	2.54	1.31	0.49	0.86	2.68	19.30
	FNS II	2,5,5,1	None	400–2500	2.60	1.28	0.41	0.90	3.13	15.79 *
	Corona	1,5,5,1	SNV + DT	500–1690	2.67	1.25	0.30	0.94	4.19	11.14
Alcohol content (% vol.)	FNS I	2,5,5,1	SNV + DT	400–2500	0.98	0.81	0.06	0.99	14.42	5.71
	FNS II	2,10,5,1	None	400–2500	0.96	0.81	0.04	0.99	22.31	3.76 *
	Corona	1,5,5,1	SNV + DT	1100–1690	1.09	0.99	0.20	0.96	4.98	18.21

Note:

^1^ Mean of the calibration set;

^2^ Standard deviation;

^3^ Standard error of cross-validation;

^4^ Coefficient of determination of cross-validation;

^5^ Ratio SD/SECV;

^6^ Coefficient of variation;

* The best of the best equations for each parameter and instrument tested.

**Table 4.** Percentage of Montilla-Moriles PDO vinegars classified by manufacturing method. PLS-DA.

**Qualitative groups**	**Unbalanced models**			**Balanced models**		

	**FNS I**	**FNS II**	**CORONA 45 VIS/NIR**	**FNS I**	**FNS II**	**CORONA 45 VIS/NIR**
	A: 94.3%	A: 92.8%	A: 94.3%	A: 91.7%	A: 93.7%	A: 93.7%
	B: 8	B: 8	B: 6	B: 5	B: 6	B: 4
	C: 1,5,5,1	C: 1,5,5,1	C: 1,5,5,1 *	C: 1,5,5,1	C: 2,10,5,1	C: 2,5,5,1

**Manufacturing method**						

Traditional	87.5%	91.7%	91.6%	87.5%	95.8%	91.7%
Submerged	97.8%	93.5%	95.6%	95.8%	91.7%	95.8%

Note:

A Percentage of correctly classified training samples after cross validation;

B Number of synthetic variables;

C Math treatment;

* The best of the best models for the instruments studied.
